# A Treatise on Diseases of the Air Passages, Comprising an Inquiry into the History, Pathology, Causes, and Treatment of Those Affections of the Throat, Called Bronchitis, Chronic Laryngitis, Clergymen’s Sore Throat, Etc., Etc.

**Published:** 1847-08

**Authors:** 


BUFFALO MEDICAL JOURNAL

AND

MONTHLY REVIEW.

VOL. 3. AUGUST, 1847. NO. 3.

### ORIGINAL COMMUNICATIONS.

ART. I.—*A treatise on diseases of the air Passages, comprising an inquiry into the history, pathology, causes, and treatment of those affections of the throat, called Bronchitis, chronic Laryngitis, clergymen's sore throat, etc., etc.* By HORACE GREEN A. M., M. D., &c, New-York and London, Wiley and Putnam, 161 Broadway, 1846. 8vo. pp. 275.

If the commotion produced by a new work could satisfy the expectations of authorship, Dr. Green has reason to congratulate himself upon the success of the treatise whose title page is prefixed to this article. He is, at least, secure against a calamity most dreaded by authors, that of the utter indifference and silence of the public. "And what said the world to your three paradoxes," asked the Vicar of Wakefield of his son who was recounting the history of his literary career—"the world said nothing," was the reply. The world has had much to say in the present instance, but all that has been said, if the author be fastidious, cannot have been entirely agreeable. One would hardly have supposed that a small, and apparently unpretending volume on diseases of the air passages, could have given occasion for much excitement among medical readers, still less with the public generally; yet such has been the fact. The merits and demerits of the book, and more particularly of the author, have been the subject of warm discussion, not unmixed with asperity, both in medical serials and in the newspapers. To such an extent, and with such acrimony has the controversy been pursued, that a contemporary has styled it, not inaptly, the "bronchitis war." The author and his work appear to be unfortunate in both friends and opponents, the extravagant laudations of the former, having been, perhaps, not less prejudicial than the severe criticisms of the latter. We have waited with some impatience for the work which has created so much tumult, and at length we have to acknowledge its reception. We have endeavored to give it a candid examination. We purpose to present to our readers, in the first place, a synopsis of those portions which possess novelty, or are practically important; we will, next, offer such considerations as our own reflections and observations have supplied; and, finally, we will devote some little space to the personal matters in controversy. In the latter, we shall have the advantage of being able to survey the *battle field*, after the smoke and din of the conflict have in some measure subsided, and, we may be permitted to add, without participating in any local or personal prejudices, if any such have influenced either party in the contest referred to.

The title page of the work challenges some critical notice. It may be said that an author has a right to christen his literary offsprings by whatever names he chooses, and is only amenable to criticism on the score of taste. This, however, allows a latitude which may be abused. A title page, in a certain sense, is like a descriptive label attached to mercantile wares—it is intended to inform the purchaser what he is buying. Hence, it may not only be an offence against good taste, but a deception, implying that the work is what it is not. We do not attribute any intentional fraud of this kind in the present instance, yet the author has inadvertently laid himself open to the imputation. The work professes to be a "treatise upon the air passages, comprising" &c. Now this implies that it embraces the diseases to which the bronchi, air vesicles, &c., are subject; but the fact is, it has not this comprehensive scope, and is *devoted* to the affections which are said to be *comprised* in it. On the back of the volume we have *Green on Bronchitis, &c., &c.*, but bronchitis is only incidentally, and very cursorily noticed. The title page farther announces that the work comprises an inquiry into the history, pathology, &c., of "those affections of the throat called Bronchitis, chronic laryngitis, clergyman's sore throat, &c." We would inquire by what latitude of signification can *bronchitis*, be included as *a disease of the throat!* Nor is Laryngitis properly so denominated in a scientific, or tecknical sense. The burthen of the work relates to *Pharyngitis* and *Laryngitis*, but the former is not even mentioned in the title page. We presume these are faults of carelessness, but, as such, they are hardly excusable, and in no unkind spirit we take the liberty of advising the author to correct them in another edition.

The first chapter is occupied with the "*Anatomy of the* Larynx; *Trachea, and Bronchi.*" We adopt in our quotation the punctuation of the author, who places a semicolon after *Larynx*. We know no reason for this, except, it be to intimate that the Trachea and Bronchi are dismissed with a very brief description.

Chapter II, relates to the "*Physiology of the Mucous Follicles.*" These chapters contain nothing but what is to be found in common anatomical treatises.

Chapter III, "*Pathology of the Throat, Larynx, and Bronchi.*" In this chapter the author notices acute Laryngitis,and very briefly, bronchitis, in order, as he states, "to establish the proposition that there exists an abnormal condition of the throat, and the air tubes, constituting a distinct species of disease, and which, hitherto, has been confounded by writers, and medical practitioners, with other disorders of these parts, under the names of "Bronchitis," Chronic Laryngitis," &c."

We defer remarks which here suggest themselves, to present them in connection with chapter IV, the caption of which is, "*Follicular inflammation of the throat and air passages.*" Here the author repeats the statement above quoted, viz:—"That peculiar affection of the throat, which under the appellation of Bronchitis, Chronic Laryngitis, Clergyman's sore throat, etc., etc., has occurred, especially, during the last ten or fifteen years, so frequently among public speakers and others, consists primarily and essentially; as I shall be able I think to demonstrate; *in a diseased condition of the glandular follicles of the mucous membrane of the throat, larynx, and trachea.*" The point which we wish to comment upon, is the assumption of the author that *follicular disease of the throat*, as a special malady, has not been known anterior to his own observations and the present publication. Now, if we will consult Dunglison's Practice of medicine, edition of 1842, a work which has been in the hands of students and practitioners pretty generally from the moment of its emission from the press, he will find a distinct section devoted to this subject, under the head of "*follicular inflammation of the Pharynx*." The learned author just named, gives a discription of the affection, coinciding in all essential points with that of Dr. Green, stating distinctly that he refers to the malady popularly known as Clergyman's sore throat. How generally practitioners have been in the habit of recognizing the affection we cannot say; we have ourselves had our attention directed to it, more or less, for the last ten years, and, we presume, this has been the case with most practical physicians The statement, or, at all events, the intimation of originality in regarding the affection as a special, and follicular affection, which Dr. G., seems to hold out, is, then, not capable of being sustained. Perhaps we do him injustice in supposing that this inference was intended. It is true that farther on in the chapter under consideration, he states that he has been able to discover no allusion to the essential nature and characteristic features of the disease *anterior to the last twelve or fifteen years*, yet, from the quotations already made, and the general tenor of his remarks, the impression left upon the mind of the reader is, that he claims to treat of a malady which, in so far as its pathology and clinical history are concerned, is new.

The author proceeds to the history of "Follicular disease of the throat and air passages." This, after a brief description, which is included in the symptomatology subsequently treated of, and to which we shall presently refer, embraces an account of 21 cases, occupying a considerable portion of the volume. These it would be difficult to analyze within reasonable limits, and this is the less necessary, inasmuch as they do not deviate in material points from the characteristic features embodied in the general description of the affection, and the diagnosis is seldom difficult.

Chapter VI, *Pathology of Follicular disease of the air passages.* The follicles may become hypertrophied, indurated, and ulcerated.

The follicular secretion looses its bland, transparent character, and is transformed into an acrid, adherent discharge, which becomes a source of irritation, and serves to extend the disease. The author has observed hemorrhage from the pharyngeal mucus membrane, the exudation of the blood being visible, but without any rupture,or obrasion being apparent. Pus may be secreted from the Pharyngo tracheal follicles. The author thinks that tuberculous deposits are sometimes found on the surface of the membrane lining the larynx, or collected in the mucus follicles of this cavity. In this opinion he differs from Louis, and some other authorities, but is sustained by Hope, Carswell, Williams, and others.

We extract the concluding portion of this chapter, giving a description of changes which the pharynx undergoes during the progress of the affection, and after recovery:—

"One of the earliest, and most common alterations which take place in the mucous lining of the air-passages, in the early stage of follicular disease, is an increase in their thickness. Invited by the chronic irritation which has been set up in the diseased follicles, there is, at first, an additional quantity of blood received into the contiguous mucous, and sub-mucous cellular tissues. This is followed by an infiltration of serum, within the substance of these tissues, by which interstitial deposition, they are rendered swollen and pulpy.

In the more chronic, and long-continued form of inflammation, an interstitial infiltration of lymph sometimes takes place, which renders the mucous lining more dense, and constitutes the true hypertrophy of this membrane.

In almost all cases of follicular disease, however, there occurs, eventually, an opposite condition of things from the above: for, after the affected glands have poured out their increased, and vitiated secretion for a long time, not only are the surrounding, engorged membranes unloaded, and their increased thickness removed, but the sub-cellular tissues, and the pharyngeal muscles become atrophied; in part, probably, from the increased absorption which has been set up; and we then have, on inspection, those enlarged, or cavernous throats, so frequently observable in long-continued follicular disease, and to which, allusion has, more than once, been made.

Connected with, and following this morbid condition of the throat, is an interesting physiological fact, which it may bs proper here to mention. After the removal of the disease by a successful plan of treatment, a deposition of healthy, structural matter commences, and the calibre of the enlarged throat, is in a short time, greatly reduced in its diameter. The filling up of the posterior pharynx, in these cases, usually commences, first, on the right side; so that, not unfrequently, the fleshy fibres of this side, will be, for a time, increased in thickness, to a considerable extent, beyond those of the left. The muscles of both sides become at length, fully, and equally developed; until this takes place, however, perfect, and natural vocalization will not be fully restored."

Chapter VII. *Causes of Follicular diseases of the air-passages.* —The author attaches some importance to *hereditary agency.* The strumous-diathesis is supposed to prove a remote cause. Climate seems not to exert a notable influence, cases occurring at the South as well as at the North. Constitutional debility, especially if it result from severe and protracted mental labor, conjoined with mental anxiety, is a prolific cause, in the author's estimation, and to this he thinks is due the fact that it prevails so extensively among the clergy of this country. It prevails more among males than females; hence, sex may be suspected to have some agency. As regards age, it occurs generally between the years of twenty-five and thirty-five. It is apt to succeed an attack of influenza. The affection came into notice shortly after the epidemic influenza which prevailed so extensively in 1830, and was probably due, in some measure, to that epidemic. Cases have multiplied shortly after each succeeding epidemic. In its connection with Dyspepsia, Dr. G., thinks it sustains the relation of cause rather than effect. It appears in some instances to be developed by the eruptive fevers. The exercise of the voice when constant, or regular, although excessive, does not seem to induce it, but when occasional, as in the duties of clergymen, it may be regarded as a frequent cause. Lastly,the author mentiones the use of tobacco as an exciting cause offerring a powerful obstacle in the way of recovery.

Chapter VIII. *Symptoms of Follicular disease of the air-passages* —Here we shall quote somewhat at length:—

"It has been stated, in a former chapter, that the access of follicular laryngitis, is, in some instances, so insidious, and its progress so gradual, that not unfrequently, it may continue many months, and make considerable advance, before the manifestations of disease shall be such, as to alarm the individual, or to call his attention, even, to the existence of the affection.

Ordinarily, however, soon after the mucous glandulæ have taken on a morbid action, there is perceived in the region of the fauces, an increased mucous secretion; and an uneasy sensation in the gullet or upper part of the throat is observed, attended by a frequent desire to swallow, as if some object, sticking in the passage, might be removed, by the act of deglutition; or, more generally, repeated attempts are made, by hawking, to clear the throat, and allay the irritation; all which difficulties are considerably augmented by every continued effort made to read aloud, to sing, or to speak, as in ordinary conversation. If the secretion from the mucous follicles of the throat be examined at this period, it will be found to be altered in its character,—being adhesive, and, in some instances, of an alkaline quality, and proving to be, by its effect on the mucous membrane, of an irritating nature.

About the same time, if the patient be accustomed to employ the voice, in public speaking, or in singing, there is apparent to a greater or less extent, a loss of power, in the vocal organs; uneasiness in the larynx, with, sometimes, pain on pressure. Hoarseness, is also present, which may be light, in the morning, or altogether absent, but which is increased, towards evening and after speaking longer, or louder than usual.

On inspecting the throat, the fauces and the posterior wall of the pharynx will appear redder than natural; and the mucous membrane covering these parts, will be deprived of its epithelium, injected, and studded over with enlarged mucous follicles. Sometimes, if the disease is recent, these glands will appear quite minute, and will be distinctly apparent, only, when the pharyngeal cavity is exposed to a full light. In other instances, they will have attained a size, sufficient to give a rough, or granular appearance, to the whole surface of the fauces; while the viscid, tenacious mucous, which is poured out by these follicles, in their morbid state, may be seen, coating the membrane, or appearing in patches, or marking its surface with white, or yellowish white striæ.

In some cases, several of the enlarged and morbid cryptæ will become confluent, and uniting, form angry looking tubercles, of the size of a split pea, which may be seen on the posterior wall of the pharynx.

In others, again, a deposition of textural matter takes place, and the follicle becomes indurated, and permanently enlarged; or it may be distended with pus, or with a morbid secretion which will exhibit all the physical properties of tuberculous matter.

If the affection has continued for some time, we shall frequently find some of the diseased follicles in an ulcerated state; these are generally first observed about the palatine arch, the posterior wall of the pharynx, and along the border, and on the laryngeal face of the epiglottis. In the first stage, these ulcers are small and superficial,—appearing in the form of ash-colored patches, surrounded by an inflamed, and slightly elevated base. Continuing, they at length destroy the mucous follicles; and, sometimes involve, not only the mucous, but the sub-cellular tissues in their progress.

Accompanying the above symptoms there are often found œdema and elongation of the uvula; and, in many instances, hypertrophy of the tonsils.

If the patient be exempt from all hereditary, phthisical tendencies, these symptoms may continue for years, without making any decided progress. At times, the unhealthy appearances will be nearly,or altogether absent,and will return again,whenever the individual is exposed to any of the ordinary, exciting causes. Some causes have come under my care, in which the disease,—its symptoms alternating in this way,—has continued for fifteen or twenty years; affecting only the follicles of the lining membrane of the air-passages; but, in other instances, where the disease had not been in progress, as many months, yet, where a strumous diathesis existed, I have found the lungs, in this period irremediably affected, although the disorder was entirely local in its origin, and had been limited, in its incipiency, to the pharyngo-laryngeal cryptæ."

Mental depression, the author has observed so constantly, that he is almost led to consider it characteristic of the disease. In this respect the state of mind contrasts strongly with that peculiar to Phthisis.

As regards ulceration about epiglottis, we extract as follows:—

"The existence of erosions, or superficial ulcerations about the epiglottis, in throat-ail, is a lesion, much more frequent, in its occurrence, than is generally supposed. In a large proportion of the cases of follicular laryngitis, which have come under my care, where the affection has been long continued, I have found more or less of ulcerations of the cryptæ of the epiglottis. These erosions are frequently found occupying the edge, or border of this fibro-cartilage ; and they may, often, be seen, distinctly, by pressing down the base of the tongue, with a broad crooked spatula. By the same movement too, I have discovered, not unfrequently, deep and ragged ulcerations, burrowing in the fossæ, which are situated below the lenticular papillæ, at the base, or roots of the tongue, where the attachments between this organ, and the epiglottis exist. Ulcerations, occupying the latter position, are productive of much mischief; and, from their peculiar position, are very likely to escape detection. Unless great pains are taken, to draw the whole mass of the tongue, downwards and forwards, their situation will not be observed. Being protected by the abasement of the tongue, unless this organ is drawn forward, and depressed, in the manner just described, they are not, ordinarily, reached, by the topical application, in the attempt to cauterize the throat.

The symptoms which characterize the presence of these lesions, do not differ, essentially, from those which indicate the existence of ulcers in the laryngeal cavity; they are, soreness, on one, or both sides of the throat, just under the cornua of the os hyoides; hoarseness, often, with more or less cough and expectoration of an opaque secretion—sometimes free—which seems to come from the opening of the wind-pipe, or very top of the throat. This expectoration is, frequently, increased after eating, and is sometimes, tinged with blood; or small masses of dark, almost coagulated blood, will be mingled with the sputum. Several cases have come under my notice, during the present year, where, topical measures having been employed, until the laryngeal affection had disappeared, these symptoms have remained, and have been found to depend on the existence of old ulcerations, in the above described locality."

The following is a description of the different appearances which the epiglottis may present:—

"The effects produced on the appearance and position of the epiglottis by ulcerations of its follicles, and of those of the larynx, are so uniform, under similar circumstances, that I have been accustomed to view the different aspects, presented by this organ in disease, as in some degree, characteristic of the location and extent, of the internal organic lesions. The appearances consequent upon these ulcerations, differ according to their seat and extent; but my observations have not been sufficiently extended to enable me to arrange, and classify, the facts obtained, with that precision which I hope,—by calling the attention of the profession to this subject,—may yet be accomplished, when more materials shall have been collected.

If the follicles, situated along the border, and on the laryngeal face of the epiglottis, become ulcerated, I have observed, that this organ, which in its normal state is slightly crescentic, loses this form, and appears flattened like the tongue; it is moreover, enlarged and thickened, and its border may be seen, frequently, serrated by the erosions.

When the cluster of follicles, which constitutes the epiglottic gland, becomes the seat of ulceration, the epiglottis will assume nearly an erect form, and be found incurvated, or its crescentic shape considerably increased; and when this lesion hss extended to the numerous glandulæ of the ventricles, and to those around the chordæ vocales, the above alteration of the form of the epiglottis, will be still greater; its lateral edges, will then be found rolled in towards each other, so that the organ will present, nearly a tubular form, with its convexity towards the dorsum of the tongue."

Finally, the affection may exist as a complication of Bronchitis, and of Phthisis. The latter affections are to be determined by their proper symptoms and signs. Dr. G., however, is of opinion, that, under these circumstances, life may frequently be prolonged, and sufferings mitigated by the employment of topical remedies adapted to the laryngeal irritation.

Chapter IX. *Of the treatment of Follicular Disease.*—The remedy upon which most, and, indeed, the chief reliance is to be placed, is the nitras argenti, topically applied in solution. The following is the course recommended:—

"*Method of applying the solution.*—In the treatment of laryngeal disease, by the direct application of the nitrate of silver, to the diseased surface, I have employed, ordinarily, a solution of this substance, of the strength of from two to four scruples of the nitrate, to an ounce of distilled water. When, however, there are found extensive ulcerations of the epiglottis, or, about the opening of the larynx—ulcerations which it is desirable to arrest at once, I have not hesitated to apply directly, to the diseased parts, a solution of double the strength of the last named. But, one or two applications, only, of a medicine of this power should be made, at one time; ordinarily, however extensive the lesions may be, it will not be necessary to employ a solution of greater strength, than one composed of four scruples of the salt, to an ounce of water. On the other hand, it has been found, that one of less strength than of from forty to fifty grains of the nitrate to an ounce of fluid, will have but little effect upon a diseased mucous surface, where ulcerations exist.

In cases in which it becomes necessary to cauterize the interior of the laryngeal cavity, the aperture of the glottis should not be passed at once; the part should be *educated*, by applying the solution daily, for several days, to the faucial and pharyngeal region; to the epiglottis, and about the opening of the glottis.

Proceeding in this manner, that exquisite sensibility which belongs to the lips of the glottis, is in a good degree, overcome, and the instrument may then be passed into the larynx, without producing half the amount of that irritation which if introduced below the epiglottis, it would have awakened at first.

The instrument which I have always employed for making direct, medicinal applications into the cavity of the larynx, is one composed of whalebone, abont ten inches in length; (with, or without the handle, as represented in the plate) curved at one end, to which is securely attached a small, round piece of fine sponge.

The extent to which the rod is to be bent, must be varied according to circumstances; for the opening of the glottis is situated much deeper in some throats, than in others; but the curve which I have found suited in the greatest number of cases, is one which will form the arc of one quarter of a circle, whose diameter is four inches.

The instrument being prepared, and the patient's mouth opened wide, and his tongue depressed; the sponge is dipped into the solution to be applied, and being carried over the top of the epiglottis, and on the laryngeal face of this cartilage, is suddenly pressed downwards, and forwards, through the aperture of the glottis, into the laryngeal cavity.

This operation is followed by a momentary spasm of the glottis, by which the fluid is discharged from the sponge, and is brought into immediate contact with the diseased surface.

Every physician who has been present when this operation has been performed, (and a large number have witnessed it, from time to time,) has manifested much surprise on observing how little irritation has been produced by the introduction of the sponge.

If the patient, on opening his mouth, take a full inspiration, and then be directed to breathe gently out, at the moment in which the sponge is introduced, the irritation, caused by the application, will be much less, than when this caution is not observed. The fact, indeed, has been fully established, by repeated experiments, that the introduction into the larynx, of a sponge, saturated with a solution of the crystals of nitrate of silver, of the strength of forty, fifty, or even sixty grains of the salt, to the ounce of water, does not produce, ordinarily, as much disturbance as is caused by the accidental imbibition, into this cavity, of a few drops of tea, or even of pure water!

In the topical treatment of the follicular disease, it will be found, that all larynges cannot be entered with the same facility. Indeed, in some instances, where œdema of the epiglottis, and of the arytenoid cartilages, has existed, I have found it very difficult, in making the first attempt, to pass the sponge of the probang through the aperture of the glottis."

The following extract, in connection with the foregoing, embraces all the important practical considerations connected with this part of the treatment:—

"In the simple and uncomplicated form of follicular pharyngo-laryngeal disease, however severe the local affection may have been, this remedy, alone, namely: the crystals of nitrate of silver, topically applied, has proved, in my hands, a specific in a large number of cases. Its use, when the affection has been of long standing, should be continued for some time. Ordinarily, it is better to make the applications, at first, every other day, for two or three weeks; subsequently, twice or three times a week, until the granular, and vascular mucous surface assumes a smooth, and healthy appearance, and impaired vocalization is fully restored.

Instances will occur where several of the enlarged, and diseased follicles of the throat will become confluent, and present angry-looking tubercles, which may be seen on the pharyngeal membrane, below the palatine arch. These should be touched, occasionally, at first, with the solid nitrate, and the solution subsequently be employed.

Not unfrequently will the mucous cryptæ in the posterior nares become involved in the disease; and when this occurs there will be an almost incessant dropping down, into the throat of unhealthy mucus from this cavity. On several occasions I have found old ulcerations of the follicles of the fossæ, from which a most offensive secretion was constantly percolating.

This diseased condition of the lining membrane of the fossa nasalis, is more certainly relieved by the topical application of the nitrate to the affected parts, than by the employment of any other known treatment.

Applications to the nasal cavity are readily effected by means of a small rod of whalebone, which, instead of being curved, should be bent at nearly a right angle, one and a half inches from the end, and be armed with a small thin piece of sponge. This, being dipped in the liquid, is carried up behind the velum, and the whole lining membrane of the posterior nares, in this way, may be sponged with the solution.

Elongation of the uvula frequently co-exists with the affection. When this occasions irritation by coming into contact with the epiglottis, its extremity should be excised. The tonsils, also, are occasionally found enlarged, and should be removed by excision.

Although the disease, in the author's view, is primitively follicular, it may extend itself so as to induce inflammation of the entire membrane. Affecting the larynx, it then becomes, in the author's language: "follicular disease complicated with chronic laryngitis." He adduces several cases to illustrate the happy effect of the topical application of a strong solution of the nitrate of silver under these circumstances.

When complicated with well marked tubercular disease of lungs, as already remarked, the topical applications, in the manner described, frequently relieve the cough, and other distressing symptoms. The applications, therefore, are quite appropriate, notwithstanding this fatal complication.

Chapter X. In this concluding chapter the treatment of follicular disease is continued, the subject being the general remedies which the author has found serviceable. When the affection is connected, as it sometimes is, with the follicular gastric dyspepsia of some writers, the nitrate of silver, internally, is superior to any other, in the author's estimation.

Iodine, when evidencies of a disorded condition of other parts of the glandular system co-exist, will be useful. The author prefers the iodide of potassium. The iodide of iron has disappointed his expectations.

Mercury is recommended, the bi-chloride being preferred, which, if the subject be irritable, should be conjoined with opiates.

The prussic acid he has found occasionably serviceable. So, also, the sanguinaria, united with tincture of opium, or morphine.

The muriate of ammonia, in combination with squill and digitalis has appeared useful in allaying irritation, and promoting expectoration. Finally,the work concludes with some remarks on the influence of a malarious climate as a prophylaxis of phthisis. On this subject the author has formerly published some observations. This subject, however, relates to phthisis, rather than to the affection which has been considered, and is referred to for the purpose of inviting attention to it chiefly in that connection.

We have thus endeavored to present, in a condensed form, an abstract of the practical portions of Dr. Green's work. With the indulgence of our readers, we will devote a little space to some remarks based on our own practical acquaintance with the affection. While we do not profess to have had extensive opportunities for observation, nor to have devoted to it special attention, we have, nevertheless, as already stated, been in the habit of meeting with it, more or less,as it occurs in general practice, and we submit such views as we have been led to form, that they may be taken for what they are worth. In this division of the article we will not assume enough to amplify at much length; but shall consider brevity due, at least, to ourselves, if not to the reader and the subject.

The study of specialities, with its numerous and great advantages, is not without its evils. And of the latter is its tendency to represent subordinate affections under too exclusive an aspect, shutting out of sight their dependencies and relations, exaggerating their prevalence and scope, and magnifying their importance. The *specialtist* (if we may coin the term) and they who imbibe more or less of his views and enthusiasm, are apt to see but little beyond the disease which has enlisted their interest: they see it on all sides, where, before, its very existence had not been suspected; and whenever they behold it, all else is merged in that which so entirely absorbs their attention. Hence it is, that there is some truth in the remark, that diseases, like the conventionalities of life, are subject, in a certain sense, to the mutations of fashion. Those which at any particular period take precedence as special objects of interest, derive thereby a fictitious predominance and importance. It has been somewhat so with the affection under present consideration, and the prospect is that it will be still more so for some time to come. Patients will be found to suffer from *follicular affection of the throat*, who, whilom, would have readily escaped all imputation of the kind, and who would be equally fortunate or unfortunate, as the case may be, at some future period. We doubt not sponges saturated with sol. nitras argenti will be plunged into not a few *pharynges*, and, possibly, *larynges*, which it were much better should be left unmolested, and in fact, cases of this description have already fallen within our knowledge. This bids fair to be the fashionable affection, both with the profession and the public; but we would not be understood to say, that it is, in our view, entirely an imaginary complaint. It has doubtles been formerly overlooked, imperfectly appreciated, and either neglected or maltreated; but its prevalence, and its importance, in many cases, for the reasons which we have alluded to, are likely to be overestimated.

Our attention, in the cases which have fallen under notice, has been directed to the affection generally as incidental to other disorders—for the most part disorders of a general character, affecting body and mind, which, heretofore, were classed under the comprehensive title of dyspepsia, and which we have, of late, oftener ranked under the denomination of spinal affection. We can recal but few instances in which it has not been involved with constitutional ill health, and we have regarded it as sustaining to the general state of the economy the relation of an effect, much oftener than of a cause. We do not, as yet, perceive any good reason for changing this opinion. In our experience it has usually been connected with habitual indigestion, muscular delibity, depression of spirits, &c., symptoms denoting difficulties more general, which have preceded this local affection.

It is a surprising fact that chronic pharyngitis,under these circumstauces, becomes developed imperceptibly, and not infrequently has hardly arrested the patient's attention sufficiently to have any difficulty of the throat suspected previous to its discovery by the ocular inspection of the physician. In these cases, by careful examination of symptoms, after the discovery is made, we can generally satisfy ourselves that it has existed for months, and, perhaps, for years. In accordance with the foregoing views, we must express doubts whether it has ordinarily much claim to be regarded as a special or independent affection.

Is the affection primitively and essentially a follicular disease? We are not prepared to express an opinion in the negative, but the affirmative does not appear to us to be established. The observer must not expect to find in all instances the follicles so prominently developed as they are figured in the plates contained in Dr. Green's treatise. In the cases which have fallen under our observation the pharyngeal mucus membrane has exhibited diffused redness, conjoined with a granulated appearance, which is perhaps due to the follicles. The latter, however, appears only to participate in a general inflamed condition of the tissue. Prof. Dunglison compares the affection to acne. The similitude, if just, is most with that variety called *acne rosacea* in which the morbid state of the cutaneous vessels, as well as of the follicles, enters uniformly and essentially into the disease. The term *granulated*, in our view, expresses the morbid appearance. It resembles the granular form of inflammation which affects the conjunctiva, and, in the female, the cervix uteri; and it has occurred to us whether it does not in more points than one sustain an analogy to these affections. Like them it is peculiarly persistent and obstinate; and the local remedy which they require is peculiarly applicable to it. Perhaps it may be added, that they resemble each other in being generally associated with constitutional disorder. The expression *granular pharyngitis* strikes us as a better title than *follicular inflammation*, while it remains a matter of doubt whether the follicles are the point of departure for the disease, or necessarily involved in all cases.

The affection, probably, commences in all cases with the pharyngeal mucus membrane. In the larger proportion of cases we suspect the larynx is affected only by contiguous sympathy, or by some extension of irritation into that cavity. We do not deny, however, that the inflammation, follicular or otherwise, may extend itself into, or originate within the larynx

As to the practiabilty of depressing the tongue so as to bring the epiglottis into view, we have only to say that we have been less fortunate than those who have found it easy or even possible

Why does the affection prevail so extensively among clergymen? We should perhaps preface this inquiry by asking if it be a fact that it does prevail, *par excellence*, among that class. This may be more apparent than real. The duties of the clergy eminently requiring the exercise of the vocal organs, the affection is a source of inconvenience, which is not felt by those who do not engage in public speaking. Hence, with the latter, the disease may for a long time escape attention; and, when detected, it does not incapacitate for ordinary avocations. The clergyman, on the other hand, if the difficulty be excessive, and, at all events, from apprehension that it may become so, feels compelled to relinquish his duties: he is placed *hors du combat*, for, with him, the vocal organs are his weapons, without which he can do no service. On this account the affection has, for him, a serious character much beyond its intrinsic gravity. He feels it vastly more than the layman, and it becomes also in his case much more observable by others. In this way, may, doubtless, be explained much of the apparent prevalence of this complaint among the clergy. Another reason is, the malady is sometimes supposed to exist when it does not. The habit and pursuits of the clergy tend to develop dyspeptic hypochondriasis; and, of late years, since the "*clergyman's throat ail*," has been in vogue, it has, in not a few instances, been wholly imaginary, on the difficulty has been greatly exaggerated in the estimation of patients. There is a species of mock-pharyngo-laryngitis, in which there is little, if any actual inflammation, follicular, or otherwise, but a feebleness and irritability of the laryngeal muscles in common with the muscular system in general, sufficient to create apprehension, andinduce a firm conviction, in his mind, that he is a martyr to the fashionable complaint, perhaps sufficiently to render an European tour imperative!

That there are causes peculiarly operative upon the clergy we do not deny. One of these is the custom of using the vocal organs on one day only of the seven, or, at least, in a much greater degree on the Sabbath, than on other days. It is probable that the same amount of exercise of the voice daily, would be attended with no injury. Among the clergy we do not find the ailment most prevalent among those who speak oftenest, or most vociferously—for example the methodists. But we are disposed, with the author, to attach the chief agency to the manifold causes affecting the *physique* and the *morale* of the clergy, rather than to any causes operating directly upon the organs affected.

A few words on the subject of treatment. As regards the topical application of the nitrate of silver, it is generally agreed that it is the most efficient local remedy we are at present acquainted with. In some instances its application to the pharynx will produce decided and speedy relief, but this has not been invariable in our experience. Some cases will require it to be continued for a long time, the patient deriving but little apparent benefit from the individual applications. We shall not venture any dogmatical assertions respecting the feasibility of carrying the sponge into the laryngeal cavity. We must confess, that we are somewhat in doubt on this vexed question. We have made some trials, and have produced irritation of the epiglottis, or glottis, with the sponge, or liquid, sufficiently to create spasmodic coughing with a transient sense of suffocation, but we are not satisfied that we have ever succeeded in fairly penetrating the larynx. We are free to state our skepticism of its being so easy a manœuvre as represented in the work of Dr. Green. The spectator at an exhibition of the operation may be deceived, and the operator may deceive himself in this matter. It is not easy to demonstrate that the sponge actually enters the larynx; we must judge from the symptoms produced, and these may be delusive. We are not willing to concede that this point is fully settled, but must regard it still *sub judice*, notwithstanding the many testimonials which Dr. Green offers.

Dr. Green does not, in our opinion, dwell with sufficient emphasis on the constitutional treatment to be pursued in this affection. His therapeutics appear to be too entirely surgical. Agreeably to the views we have expressed, it is usually an effect, or, at least, a coincident of other, and more general disorders ; and if the latter are not attended to, topical remedies will not be likely to prove permanently efficacious. A class of remedies which in our hands has appeared highly serviceable, Dr. G., does not even allude to. We refer to chalybeates. Some of the ferruginous remedies, or several in succession, continued for several weeks, will be found in a large proportion of cases very useful. External irritants we believe are of little or no avail. Well regulated diet and regimen are essential, and upon these the author does not dwell.

With these brief, desultory remarks, we will proceed to devote a few paragraphs to the matters at issue between Dr. Green, and the critics who have preceded us. The chief distinguishing feature of Green's book, as our readers will have perceived, consists in the advocacy of the practicability and advantage of topical applications within the laryngeal cavity. The application of the nitrate of silver to the pharynx is certainly not a new idea, but has been in use very generally for years past.

Now, assuming that is perfectly feasible to penetrate with the probang the larynx in the manner described and recommended in the work before us, it has been clearly enough established that the plan is not original with the author. The author, indeed, frankly admits this, but declares that he did not derive the idea from Trousseau & Belloc (French writers who had advocated previously the same method in all its details), but from a conversation with Dr, Johnson, the late editor of the Medico-chirurgical Review. This conversation occurred in 1838. Trousseau and Belloc published their work in 1837, and a translation was issued in this country in 1839. Here Dr. Green has committed an unfortunate error, in stating that the translation first appeared in 1841, but this error may be accounted for by the fact that an edition of the work was published in 1841, and Dr. G., may honestly have supposed this to have been the first. As a matter of policy, no man of common shrewdness would deliberately publish a false statement of facts with regard to a matter of that kind, for he would, of course, expect to be detected, and could gain nothing by it. We think therefore, that Dr. G., ought, in charity, only to be charged with carelessness, but, yet, it does appear surprising that as the question of priority would naturally arise in his own mind, he should not have ascertained dates more correctly, and he should have had the candor to have stated, also, when the work first appeared in France. The probability is, that if he derived the hint from Dr. Johnson, the latter was indebted for it to Trousseau & Bel!oc, since their work was published a year before the reputed conversation took place, and had doubtless been sent to Dr. J., he being editor of one of the most able and popular medical reviews of the day.

Waiving farther disscussion of errors of date, and the attribution of credit for the suggestion upon which Dr. G., commenced the operation of sponging the larynx, there can be no doubt that to Trousseau & Belloc is really due whatever of honor pertains to the discovery, assuming it to be a discovery. Dr. G., acknowledges that his views were confirmed by reading the work of these authors. But he fails to state that the details of the operation, are, in all essential particulars, the same as theirs, and to these authors, hardly any reference is made except to disclaim in debtedness to them. Candid criticism compels us to say there is apparent a great want of fairness in this respect. If the operation possesses the merit which Dr. G. thinks it does, why did he not quote the results of their experience; and why avoid all description of their methods of procedure!

Upon the appearance of Dr. G's work, attention was called to it in various literary periodicals, and in the daily papers, as containing a new discovery by the author, the importance of which was compared to that of vaccination by Jenner! We make this statement second-hand, none of the articles referred to having come under our notice. These pretensions, which Dr. G. has subsequently shown were volunteered by his too zealous friends, were not at the time promptly disowned by himself, but, as might have been expected, they awakened professional criticism. His own agency, or connivance, in procuring such extravagant eulogiums was charged upon him, but this charge, it appears to us, he has satisfactorily rebutted. That he has been too harshly dealt with by critics, and his professional brethren in the city of New-York, we are persuaded, yet we must say that he is either very peculiarly unfortunate, or he is justly open to censure for omitting proper acknowledgments to the authors who had preceded him, and anticipated most of the practical precepts of his work. The work also, we are compelled to say, bears internal evidence of having been written with a view to popular effect, a fault which should not escape severity of reprobation.

A considerable portion of it has but little relevancy to those points which are novel or important, and, without detriment to the reader, could have been dispensed with. That which is of much practical interest would admit ot being condensed into a short article,and we believe that the quotations we have given, contain all that the practitioner would desire.

Much room, however, as there is for critical animadversion, and notwithstanding the evils which we have incidentally referred to, the treatise will, as we think, in the main, be beneficial. It will serve to direct attention to an affection which is not so limited in its prevalence as its popular title implies, and which many practitioners have been in the habit of overlooking and neglecting. If it should be established that topical applications may be made to the laryngeal mucus membrane with facility, it will assuredly be instrumental in enlarging our means of treating with success affections of that part; and it is to be borne in mind, that although not the originator of this plan of treatment, Dr. Green has been (so far as we know) the first in this country to adopt it, and to advocate its feasibility and usefulness. In so far, his exertions are meritorious and important in proportion to the value, which, upon farther trial, the method shall be found to possess, and it would be unjust to overlook his claims in this point of view, in the solicitude that justice shall be done to others, and to repudiate an apparent disregard of some of the precepts of medical ethics.

